# Vacuum sealing drainage combined with naso-intestinal and gastric decompression tubes for the treatment of esophagogastrostomy neck fistula

**DOI:** 10.1186/s13019-022-01883-x

**Published:** 2022-06-13

**Authors:** Chuan Tian, Kaihao Xu, Yanan Zhao, Yahua Li, Kunpeng Wu, Dechao Jiao, Xinwei Han

**Affiliations:** Department of Interventional Radiology, The First Affiliated Hospital of Zhengzhou University, No. 1 Jianshe East Road, Zhengzhou City, 450052 Henan Province China

**Keywords:** Vacuum sealing drainage, Nutritional tube, Gastric decompression tube, Esophagogastrostomy, Fistula

## Abstract

**Objective:**

To evaluate the clinical results of the vacuum sealing drainage (VSD) combined with a naso-intestinal nutritional tube (NIT) and a gastric decompression tube (GDT) for the treatment of esophagogastrostomy neck fistula (ENF).

**Methods:**

From January 2018 to October 2020, twenty patients (13 men and 7 women, ages 46–72) with ENF secondary to esophagogastrostomy were treated with VSD combined with NIT and GDT. Technical and clinical success rates, the incidence of early/late complications, the time of fistula closure (TFC) and therapy-related indicators were analyzed. The Karnofsky score and Eastern Cooperative Oncology Group (ECOG) score were compared before and after triple treatment.

**Results:**

Technical and clinical success rates were 100% and 85%, respectively. Early complications occurred in 5/20 (25%) patients, and late complications occurred in 8/20 (40%) patients. The median TFC was 18 days (range 10–23). All therapy-related indicators were normalized posttreatment. The Karnofsky score and ECOG score after treatment were significantly different compared with pretreatment scores (*p* < 0.001).

**Conclusion:**

VSD combined with NIT and GDT is a safe and effective strategy for ENF, while severe strictures warrant further research.

## Introduction

Although the occurrence of esophagogastrostomy neck fistula (ENF) can be prevented by optimizing the surgical strategy, its incidence is still greater than 10% [1]. If the disruption is limited, ENF could be addressed with conservative treatment, including wound care, gastrointestinal decompression, jejunal or parenteral nutritional and antibiotics [2–4]. If ENF cannot be controlled well, other treatment strategies, such as stent placement or reoperation, should be applied [4–6]. If ENF extends into the thoracic cavity, a longer period of time and more treatments are required than when ENF is confined to the neck. Meanwhile, the burden on patients and the risk of death are bound to increase [3, 7].

Esophageal stent placement has been considered to be an effective nonsurgical treatment for tracheoesophageal fistula in previously published papers [8–10]. Stents provide a suitable internal environment for ENF healing with less risk than a reoperation. However, the stent is extremely easy to displace due to the special anatomy of this location, with the short residual esophagus and the stomach moving up into the chest. In addition, patients will suffer from a foreign body sensation, airway compression and a risk of stent ingrowth.

The conventional treatment of ENF is combination of a nutritional tube (NIT) and a gastric decompression tube (GDT) at our center with the aim of providing suitable internal environment to stimulate granulation tissue proliferation around the fistula. VSD is widely used for superficial or deep skin repair, the management of non-healing diabetic foot ulcers and the treatment of osteomyelitis [11, 12]. Vacuum sealing drainage (VSD), using negative pressure, has unique advantages and potential for the treatment of ENF. The site of the ENF opening to the skin provides a necessary condition for VSD. Therefore, we modified the conservative treatment strategy, creating a triple treatment that combines VSD with NIT and GDT. In this study, we retrospectively analyzed patients with ENF who received this triple treatment to explore its safety and efficacy in the treatment of ENF.

## Materials and methods

This study was approved by the Ethics Committee of the First Affiliated Hospital of Zhengzhou University (2018-ky-011), and written informed consent was obtained from each patient.

### Patients

Between January 2018 and October 2020, the data from patients diagnosed with ENF who underwent triple treatment were retrospectively collected and analyzed. The criteria for the treatment were as follows: (1) ENF confirmed by endoscopic or fluoroscopic esophagography; (2) unsuitable or failed treatment with an esophageal stent; and (3) Karnofsky scores ≥ 40 and ECOG scores ≤ 3.

### Strategy of the triple treatment

Placement of the NIT (Flocare, external diameter 3.33 mm, Nutricia, Netherlands) and GDT (the tube with guide wire for single use, external diameter 5.33 mm, Medsuyun, Lianyungang, Jiangsu; the vacuum drainage set, WEGO, Weihai, Shandong) was the first step of the triple treatment. Prior to the procedure, patients underwent fluoroscopic esophagography to reconfirm the location and size of the fistula. After local infiltration anesthesia of the nasal cavity and oral mucosa using 5 ml of 2% lidocaine, a 0.035-inch guide wire (Radiofocus M; Terumo, Tokyo, Japan) with a straight 5-French catheter (Torcon NB; Cook, Bloomington, IN) was introduced to the esophagogastrojejunum through one nostril, and then the NIT and the GDT were slowly placed along the stiff guide wire after the catheter was removed. After confirming that these two tubes were located in the appropriate position, the two tubes were fixed on the patient’s nose and cheek with butterfly adhesive, and marks were made to observe whether the tube would experience displacement in the future. Finally, the GDT was connected with a disposable gastrointestinal reducer.

**Fig. 1:**
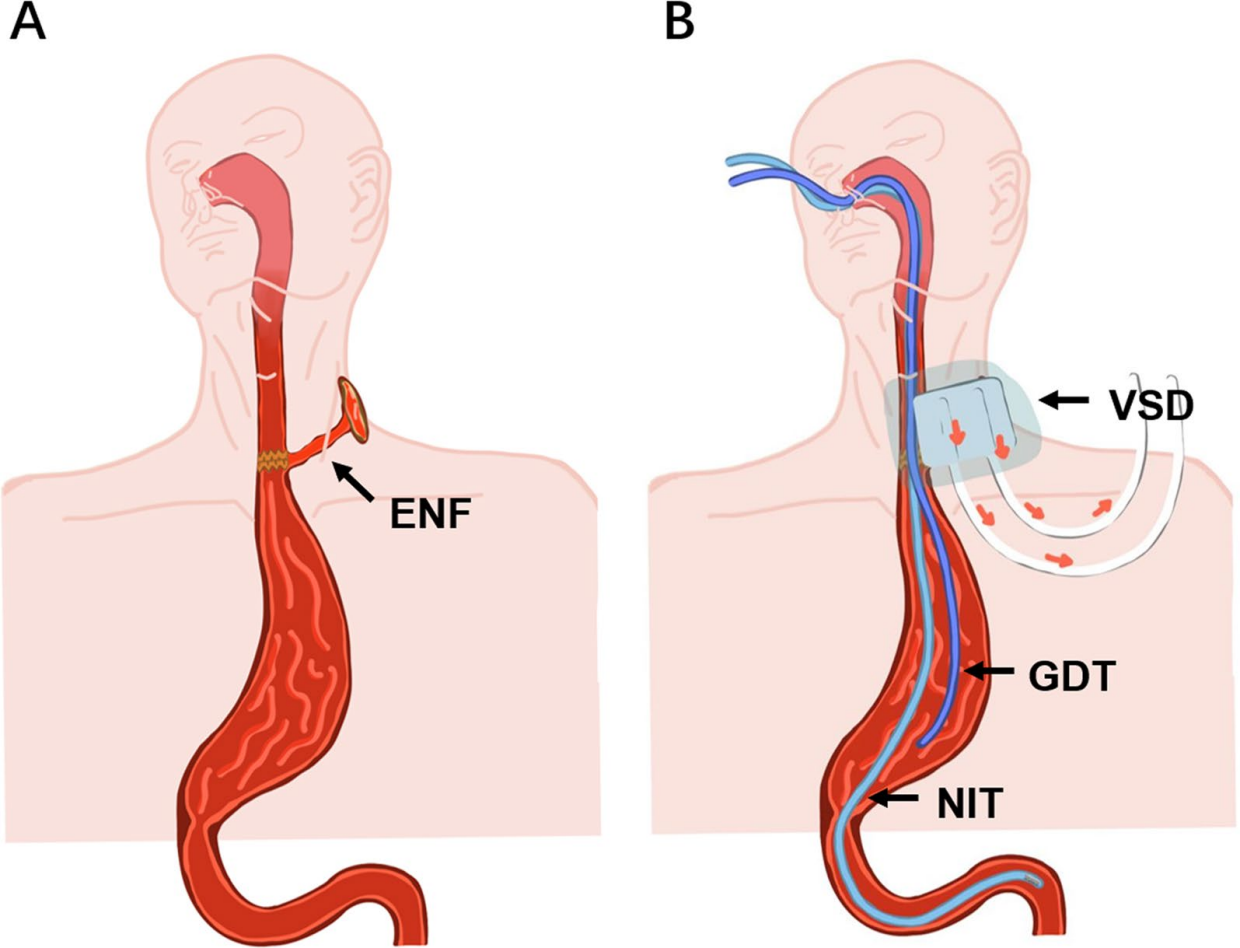
Esophagogastrostomy neck fistula (ENF) diagram (**A**) and the schematic diagram (**B**) of the vacuum sealing drainage (VSD) combined with a naso-intestinal nutritional tube (NIT) and a gastric decompression tube (GDT), that the tubes of VSD are used to connect negative pressure continuous suction

**Fig. 2:**
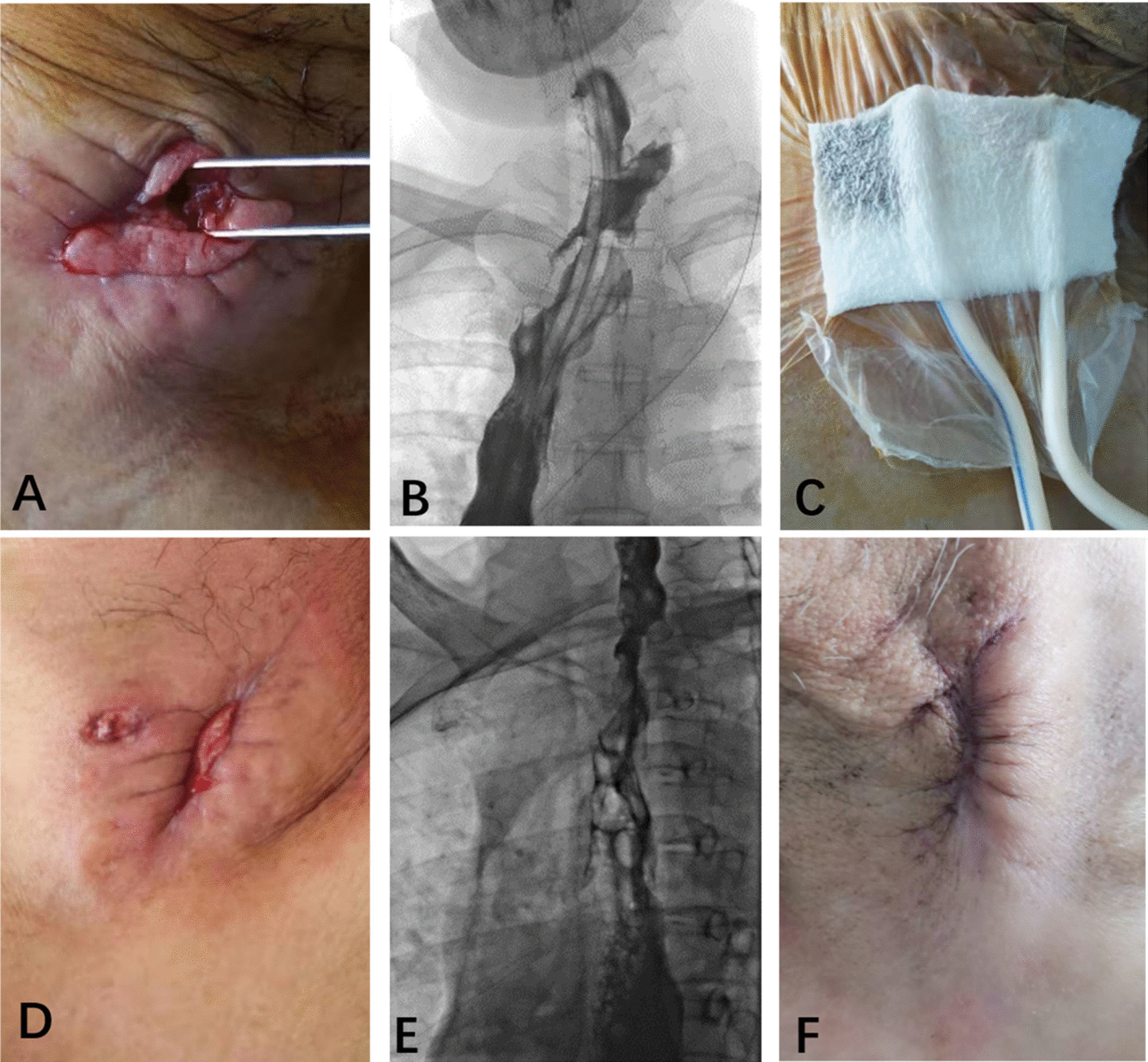
A 53-year-old male patient with ENF. **A** A skin defect was found after debridement. **B** ENF (black arrow) was confirmed by esophagography. **C** The patient accepted VSD. **D** The volume of the skin defect was decreased. **E** An esophagogram demonstrated that the ENF had closed (white arrow). **F** The skin defect was completely closed

The VSD system used in this study consists of three parts: a negative pressure source, a biological translucent membrane and a polyvinyl alcohol foam sponge dressing with two tubes: one for drainage and another for irrigation (Fig. 1). Prior to the start of VSD, debridement was performed centered on the defect using iodophor, and the disinfection diameter was greater than 5 cm. After debridement, a sponge dressing was cut according to the size of the skin defect and then adhered to the skin defect. Following this, a translucent membrane was used to cover the sponge dressing to form a completely closed environment (Fig. 2). Finally, continuous 125 mmHg vacuum aspiration was performed for 10 h and then suspended, and the patients were allowed to perform appropriate activities for 1–2 h before the vacuum was re-started. The dressing was changed every 3–5 days, and the condition of the wound was assessed at every dressing change. All those treatments were done at hospital, and the sponges were replaced by surgery team during living at hospital.

### Follow-up

The evaluation criteria for technical success were as follows: (1) NIT and GDT were safely placed in the appropriate position; (2) the VSD system provided good airtightness; and (3) the tube lumen was patent. If GDT were clogged, we used three methods to solve this problem: (1) 10ml normal saline was used injected quickly to the GDT to open it; (2) if method (1) is unsuccessful, a 0.035-inch guide wire was used to open the GDT under DSA fluoroscopy; (3) if method (2) is unsuccessful, a new GDT was replaced by a new one by DSA fluoroscopy and gastroscopy. All those manipulations were performed by interventional physicians. Clinical success was defined as reestablishment of the esophageal wall integrity confirmed by fluoroscopic esophagography or gastroscopy. The Karnofsky score and ECOG score were recorded at 1 month after treatment. The main events recorded during this study included the consumption of the sponge dressings, the time to fistula closure (TFC), the incidence of complications and the therapy-related indicators. Complications include early complications and late complications. Early complications were defined as those related to the triple treatment, such as nasal mucosal congestion, local pain, defect bleeding, eczema and the NIT+GDT tubes dislodged or pulled out (accidentally or on purpose by the patient). Late complications were defined as those occurring after treatment, such as progressive dysphagia, esophageal stricture, and the recurrence of ENF or esophageal cancer. Therapy-related indicators included white blood cells (WBCs), C-reactive protein (CRP), prealbumin (PA) and albumin. Follow-up treatments and deaths were recorded as well.

### Statistical analysis

Continuous variables are expressed as the means ± SD and ranges, discrete variables are expressed as medians and ranges, and categorical variables are expressed as counts and percentages. For data collected before or after the triple treatment, comparisons between categorical variables were performed using Pearson’s chi-square test or Fisher’s exact test, as appropriate, and continuous variables were compared using the Mann–Whitney test. All statistical tests were two-tailed, and significance was considered at *p* < 0.05. All calculations were performed with SPSS software version 21.0 (IBM Corp., Armonk, NY, United States) and GraphPad Prism 8.0.1 (GraphPad Software Inc., San Diego, CA, United States).

## Results

The demographic and clinical characteristics of the 20 patients are summarized in Table 1. The technical success rate of the triple treatment was 100% (20/20). The clinical success rate was 85% (17/20), and 3 patients could not follow a liquid diet after treatment due to NIT blockage. Those 3 patients underwent a second NIT intubation, and then could follow a liquid diet. The change in Karnofsky scores and ECOG scores posttreatment was statistically significant compared with the pretreatment scores (*p* < 0.001, Tables 2, 3).

**Table 1:** Basic-line information of patients

	Value (N = 20)	R&P
Age, years	60	46–72
*Gender*		
Male	13	65%
Female	7	35%
TAT, days	26	1–60
Length, cm	2.3 ± 0.2	1.9–2.6
Diameter, cm	1.1 ± 0.2	0.8–1.5
*Therapy-related indicators*		
WBC, 1 × 10^9^/L	7.0 ± 4.0	3.1–18.5
CRP, mg/L	68.7 ± 38.0	13.4–170.0
PA, mg/L	149.6 ± 24.6	105.8–185.0
Albumin, g/mL	37.9 ± 3.8	32.6–48.0
*Score*		
Karnofsky	56.5 ± 8.1	40.0–70.0
ECOG	2.0 ± 0.7	1.0–3.0
TFC, days	18	10–23
Sponge dressing, blocks	6	3–8
Follow-up, days	231	104–337
*Complication*		
Early complication	5	25%
Late complication	8	40%

*R&P* rang and percentage, *TAT* time to accepting treatment, *TFC* time of complete fistula closure, *PAD* polyvinyl alcohol sponge dressing, *WBC* white blood cell, *CRP* C-reactive protein, *PA* prealbumin

**Table 2:** Karnofsky score pre-VSD versus post-VSD

	Karnofsky score						Z
	40–49	50–59	60–69	70–79	80–89	≥ 90	
Pre-VSD	1(5%)	8(40%)	8(40%)	3(15%)	0	0	− 3.76
Post-VSD	0	0	2(10%)	7(35%)	9(45%)	2(10%)	(*p* < 0.001)

**Table 3:** ECOG Score pre-VSD versus post-VSD

	ECOG score				Z
	0	1	2	3	
Pre-VSD	0	4(20%)	1 1(55%)	5 (25%)	− 3.83
Post-VSD	4 (20%)	1 2(60%)	4 (20%)	0	(*p* < 0.001)

**Fig. 3:**
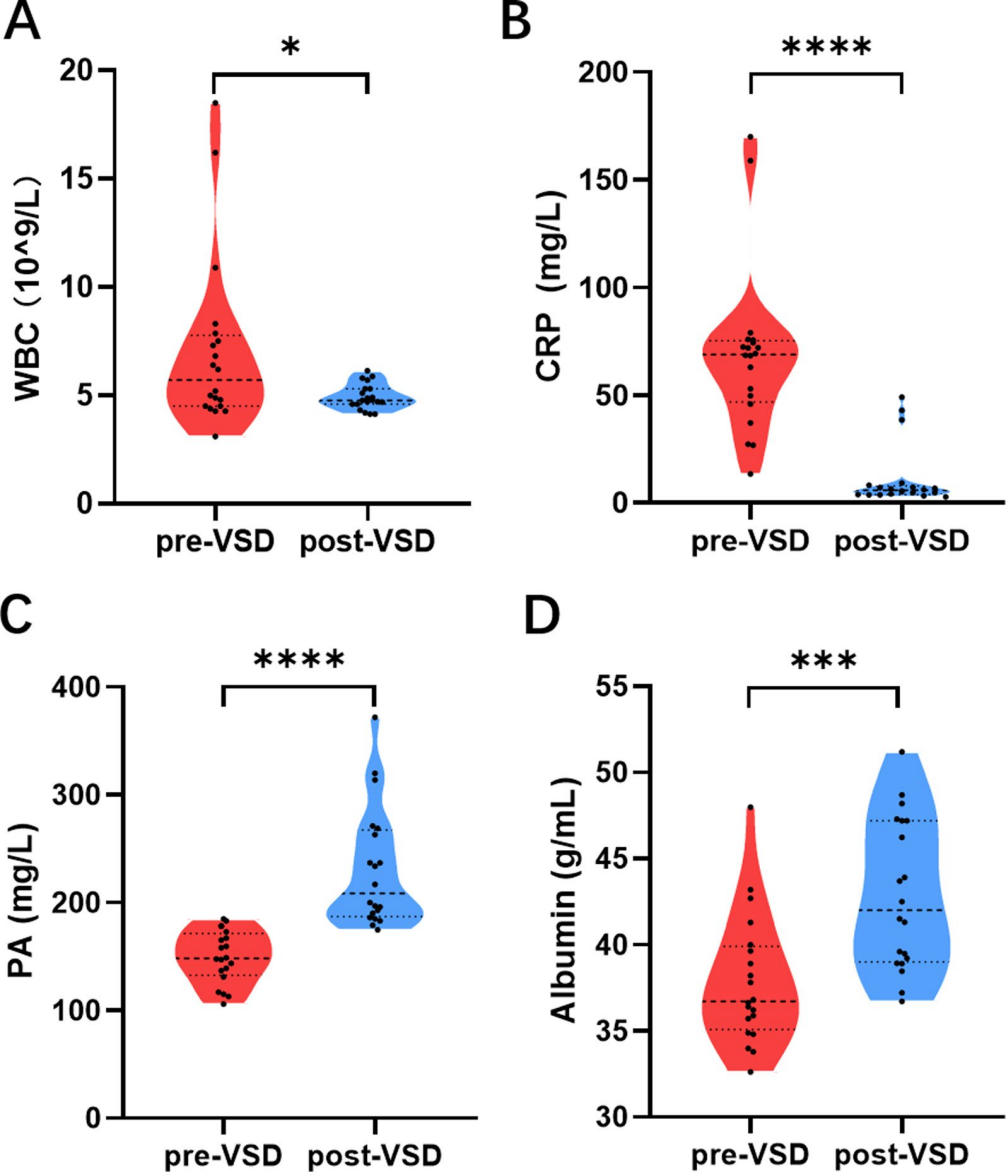
Violin plot of therapy-related indicators. **A** The WBC level was decreased posttreatment, compared with pretreatment (t = 2.35, *p* = 0.02). **B** The CRP level was significantly decreased posttreatment (t = 6.34, *p* < 0.0001). **C** The PA level was significantly increased posttreatment (t = 6.02, *p* < 0.0001). **D** The albumin level was significantly increased posttreatment (t = 3.88, *p* < 0.001). *p < 0.05, **p < 0.01, ***p < 0.001, ****p < 0.0001

In this study, a total of 111 blocks of sponge dressing were used with an average consumption of 6 blocks (median, range 3–8) per person. The median TFC was 18 days (range 9–23). Early complications occurred in 5/20 (25%) patients: local pain in 3 patients and defect bleeding with local pain in 2 patients. These symptoms resolved after adjusting the negative pressure from − 125 to − 85 mmHg. Late complications occurred in 8/20 (40%) patients: progressive dysphagia in 3 patients, esophageal stricture in 5 patients, and none patients have the recurrence of ENF. The 3 patients with progressive dysphagia were diagnosed as the recurrence of esophageal cancer through CT and pathology results, and all three patients chose conservative treatment (2 patients underwent stent replacement and 1 patient underwent NIT placement). The other 5 patients with esophageal stricture underwent multiple balloon dilatation. The WBC and CRP levels decreased significantly compared with pretreatment (*p* = 0.02 and *p* < 0.0001, respectively, Fig. 3), while PA and albumin levels significantly increased compared with pretreatment (*p* < 0.0001 and *p* < 0.001, respectively, Fig. 3). Over a median follow-up of 231 days (range 104–337), all patients were alive.

## Discussion

To the best of our knowledge, this study is the first to apply VSD for the treatment of ENF. VSD has been widely applied in clinical practice, including large area wound repair, osteomyelitis, management of non-healing diabetic foot ulcers, and burns that cannot undergo primary orthotopic skin grafting [13–15]. The principle of VSD is to connect negative pressure drainage with normal saline irrigation to ensure adequate drainage of inflammatory substances, creating a closed microenvironment for wound healing [16, 17]. Some studies have shown that adding medication irrigation, such as ozonated water and recombinant human epidermal growth factor, could accelerate wound healing [18, 19]. Animal experiments have shown that VSD can effectively promote wound healing by increasing the vessel density with hydroxyproline content, reducing the inflammatory responses and generating an ordered collagen arrangement [20]. Compared with traditional wound debridement and dressing changes, VSDs theoretically have the potential to reduce the frequency of dressing changes, shorten the healing time, and increase the success rate of wound healing.

However, VSD alone is clearly not sufficient because the esophageal defect of ENF remains exposed to an environment of saliva, food debris, and gastric juice. Therefore, we placed NIT and GDT at the same time to avoid irritation of the esophageal defect by the esophageal contents and to maximally ensure that the fistula edge environment is suitable for the generation of granulation tissue.

According to the therapy-related indicators and scores shown in Table 1, all patients showed a systemic inflammatory response, were in a state of under-nutrition and were only semi-independent before the treatment. After approximately 18 days of treatment, the WBC, CRP, PA and albumin levels were in the normal range, and the Karnofsky scores and ECGO scores showed that the patients were independent. Over a median follow-up of 231 days, all patients were alive, but 8 patients had severe stenosis. The above results suggest that this treatment is safe and effective for ENF.

However, the median TFC was approximately 17 days, which is longer than our envisaged healing time. There were three factors that were associated with the prolonged healing time in this study. First, regarding nutritional supply factors, the diet of 20 patients in this study did not strictly follow the guidance given by the nutrition department. Most patients injected liquid diet made by themselves rather than a relatively expensive commercial nutrient solution, so that an unbalanced nutrient supply to the patients will prolong the time of wound healing. Second, the time to accepting treatment was variable, and the median time was 26 days (range 1–60). The fistula environment is complex in patients with long-term inflammatory stimulation, so a longer treatment cycle for fistula closure is needed. Third, GDT has limitations in the protection of esophageal defects that cannot completely avoid any irritation from the esophageal contents due to esophageal defects. Fourth, salivary amylase of saliva is an influence factor for ENF healing. And there were some studies had shown that increased saliva level would prevent oral or esophageal fistulas from healing [21–23]. In future clinical applications and studies, the patient's nutritional supply will be strictly controlled, stratified analysis will be performed, and the protection of esophageal defects will be strengthened.

Moreover, the results of this study showed a high rate (40%) of severe stenosis after treatment. Severe stenosis reduces patients’ quality of life, and prolonged median length of stay will increase their physical, mental and economic burden [24, 25]. ENF itself is one of the independent risk factors of a stricture. Mark van Heijl et al. [26] followed 607 patients who underwent curative esophagectomy and found that the incidence of stricture after esophageal surgery was 41.7%, and in multivariate analysis, the occurrence of anastomotic leakage was an independent predictor of a refractory stricture. Except ENF itself, it is unclear if VSD or NIT+GDT could increase the incidence of stenosis. In the process of wound healing, the inflammatory promoters and inflammatory inhibitors regulated by the inflammatory microenvironment are activated or inhibited in turn, so as to regenerate granulation tissue and promote wound healing [27, 28]. However, under constant inflammatory (infectious/noninfectious) stimulation, the sequential activation of inflammatory factors will be disrupted, leading to fibroblast proliferation and differentiation and massive extracellular matrix deposition lead to granulation tissue proliferation and progressive scarring [29, 30]. The scar tissue will cause centripetal or contractile stenosis of physiological lumen [31]. According to advantages of VSD mentioned above, VSD could effective reducing the infectious inflammatory responses that is helpful for orderly regulation of wound healing, so we though that the influence to stricture of VSD is minute. The presence of two tubes of NIT+GDT may be the relatively immense influence factor for stricture. Because the mechanical irritation of tubes to ENF could cause noninfectious inflammatory responses, so that increase the risk of stricture. Inspired by the effective treatment of balloon dilation to dissolve esophageal stricture, the NIT or GDT combined with a balloon have potential to reduce the risk of strictures through avoiding the mechanical irritation and remodeling anastomotic persistently [32, 33]. The feasibility, safety and effectiveness of the balloon-carrying NIT or GDT will be investigated in our further study.

Overall, the satisfactory treatment outcome in our study demonstrated that VSD combined with NIT and GDT is a safe and effective strategy to treat ENF through negative pressure drainage, internal nutrition, and gastrointestinal decompression. However, the high stenosis rate suggests that the prevention of stenosis needs to be a focus of a future study. The small sample size and lack of controls are limitations of this study, so more studies are needed to support our conclusions.

## Data Availability

The clinical data were obtained from the interventional department of the First Affiliated Hospital of Zhengzhou University. The data used to support the findings of this study are available from the corresponding author upon request.

## Abbreviations

VSD: Vacuum sealing drainage
ENF: Esophagogastrostomy neck fistula
NIT: Nutritional tube
GDT: Gastric decompression tube
ECOG: Eastern Cooperative Oncology Group
TFC: The time of fistula closure
WBC: White blood cell
CRP: C-reactive protein
PA: Prealbumin
TAT: Time to accepting treatment
PAD: Polyvinyl alcohol sponge dressing
